# Cross-protection studies using vGA08 and Mass type IBV vaccines against four different antigenic variant viruses currently circulating in poultry in the USA and Canada

**DOI:** 10.1186/s13567-026-01718-w

**Published:** 2026-03-15

**Authors:** Mark W. Jackwood, Robert Beckstead, Jimmy White, Adrian Bustamante, Sean Brimer, Marshall Putnam

**Affiliations:** 1 Veterinary Services, Ceva Animal Health, Lenexa, KS 66215 USA; 2 US Innovation and Development, Ceva Animal Health, Lenexa, KS 66215 USA; 3 Scientific Support and Investigation Unit, Ceva Animal Health, Lenexa, KS 66215 USA

**Keywords:** Cross-protection, infectious bronchitis virus, vaccine, variant viruses

## Abstract

Infectious bronchitis virus (IBV) is a highly contagious gammacoronavirus that causes disease in chickens, typically presenting with respiratory signs but in some cases affecting the reproductive or renal systems. Due to different antigenic variants co-circulating in poultry with limited availability of homologous vaccines, producers have historically combined different vaccine types to provide a broader cross-protective immunity. This study evaluated whether two USDA licensed vaccines (vGA08 and Mass types—administered simultaneously at one day of age) can protect against challenge with 4 different currently circulating variant IBV strains in the US and Canada (PA/1220/98, CA/1737/04, NC/DARK/23, and Canada/DMV1639/23) at 4 weeks of age. For the vaccinated/challenged birds, clinical signs were reduced and the level of challenge virus detected was significantly lowered for each of the challenge viruses compared to non-vaccinated birds challenged with the same virus. This study is important because it provides poultry veterinarians with information on the level protection that can be expected using safe and efficacious commercially licensed vaccines against 4 currently circulating variant IBV strains.

## Introduction

Avian infectious bronchitis virus (IBV) is a gamma coronavirus that causes a highly contagious upper-respiratory disease, resulting in airsacculitis, production losses, and condemnations at processing. Some strains can replicate in the kidneys causing interstitial nephritis and flushing. Additionally, IBV has been associated with testicular lesions resulting in decreased fertility in breeding males. When female chicks are infected within the first 2 weeks of life, the virus can damage the immature oviduct, resulting in false layer hens that look normal but fail to lay eggs.

The disease is difficult to control, because there are many different antigenic variants and few available vaccine types. Thus, producers have historically combined different vaccine types to induce a broader, cross-protective immunity. The objective of this study was to determine if two USDA licensed, commercially available vaccines; variant GA-08 (vGA-08) in lineage GI-27 and Mass in lineage GI-1 [1], given simultaneously at 1 day of age can protect against 4 different IBV variant viruses currently circulating and causing problems in the US and Canada [2].

The challenge strains of IBV used in this study consist of two viruses that were identified some time ago (PA/1220/98 and CA/1737/04) but continue to circulate in commercial poultry as well as two more recently identified viruses (NC/DARK/23 and Canada/DMV1639/23). The PA/1220/98 in genotype GVIII [1] virus was first detected in commercial layers in Pennsylvania in 1998 [3] and has since been reported in Nebraska, Iowa, Alabama, and Pennsylvania. The CA/1737/04 virus in lineage GI-25 [1] was first detected in California broilers in 2004 [4] and has also been detected in Arizona and Canada. The NC/DARK/23 virus, a recombinant between DMV1639 (GI-17) and Arkansas (GI-9) [1], was detected in North Carolina broilers in 2023 with the majority of the S1 glycoprotein (aa 53-541) similar to DMV/1639 [2]. Although not widespread, NC/DARK/23 is a unique variant that warrants further investigation. The DMV/1639 (GI-17) [1] virus which has circulated in the US since 2011, was first identified in Canada in 2015 [5] and is now the most prevalent IBV type causing disease in Canada [6]. The Canada/DMV1639/23 virus used in this study was obtained from broilers with clinical signs of IBV in Canada and isolated in our laboratory in 2023. Its S1 amino acid sequence is 96.5% similar to US DMV/1639 strains [5].

## Materials and methods

### Ethical statement

All the animal experiments conducted in this study were in compliance with the relevant provisions in the 9 Code of Federal Regulations guidelines (I.A.2.C. 2.31-2.38, and I.A.3.G. 3.150-3.168), and with permission from our internal Institutional Animal Care and Use Committee (IACUC). All animals were euthanized by a method provided in the American Veterinary Medical Association Guidelines on Euthanasia, June 2007 and determined appropriate by the attending veterinarian.

### S1 spike sequences

The S1 spike gene and deduced amino acid sequences for the viruses examined in this study were either previously sequenced [2] in our laboratory (NC/DARK/23 and Canada/DMV1639/23) or obtained from the National Library of Medicine, National Center for Biotechnology Information [7] GenBank database. The sequences were aligned using Muscle in the Geneious software package, Version 2024.0.5 [8] and a phylogenetic tree was constructed with the Neighbor-Joining tree building method.

### Chickens and housing

Ninety specific pathogen free (SPF) chickens were obtained from AVS Bio (Wilmington, MA, USA) and held in isolators on raised grate flooring. Water and feed were provided ad libitum*.*

### Vaccines and challenge viruses

vGA08 (IBron, Ceva Animal Health) and Mass (IMass, Ceva Animal Health) were used in this study. The pathogenic challenge viruses used were PA/1220/98, CA/1737/04, NC/DARK/23 and Canada/DMV1639/23 obtained from our repository.

### Reverse transcriptase-quantitative polymerase chain reaction (RT-qPCR)

A universal 5UTR assay, which detects all IBV types and type specific RT-qPCR assays that detect the vGA08 and Mass vaccine viruses as well as the DMV/1639 challenge virus were conducted as previously described [9, 10].

### Experimental design

Forty SPF chickens were vaccinated at 1 day of age (DOA) with the manufacturers recommended dose of vGA08 (IBron, Ceva Animal Health) and Mass (IMass, Ceva Animal Health) by eye-drop. Forty additional birds were not vaccinated and served as positive challenge control birds, and 10 birds were not vaccinated and served as negative controls (no challenge).

At 5 days post-vaccination (dpv) the choanal cleft of each bird was swabbed and tested by reverse transcriptase- quantitative polymerase chain reaction (RT-qPCR) using a universal 5UTR assay as well as specific RT-qPCR assays for the presence of the vGA08 and Mass vaccine viruses [9, 10]. At 26 dpv, all birds were swabbed via the choanal cleft and bled via the wing vein and tested for vaccine virus using vGA08 and Mass type specific RT-qPCR assays and for antibodies against IBV by the ELISA test (IDEXX Westbrook ME) respectively. Birds in the challenge groups were challenged at 28 dpv with either; PA/1220/89 (1 x 10^2.0^ EID_50_/dose), CA/1737/04 (1 x 10^4.0^ EID_50_/dose), NC/DARK/23 (1 x 10^3.7^ EID_50_/dose) or Canada/DMV1639/23 (1 x 10^4.0^ EID_50_/dose). At 5 days post-challenge (dpc), birds were examined for clinical signs and scored as previously described [11] and the choanal cleft was swabbed for detection of challenge virus. For the birds challenged with NC/DARK/23 and Canada/DMV1639/23, the DMV/1639 type specific RT-qPCR test was used. And since type specific tests were not available for the PA/1220/89 and CA/1737/04 challenge viruses, birds challenged with those viruses were tested using the universal 5UTR RT-qPCR test.

### Statistical analysis

The clinical sign scores were analyzed by the Kruskal–Wallis test followed by Tukey multiple comparison test. Student’s *t*-test was used to analyze the amount of challenge virus detected by RT-qPCR. The data were analyzed using GraphPad [12].

## Results

### Sequence analysis

**Figure 1:**
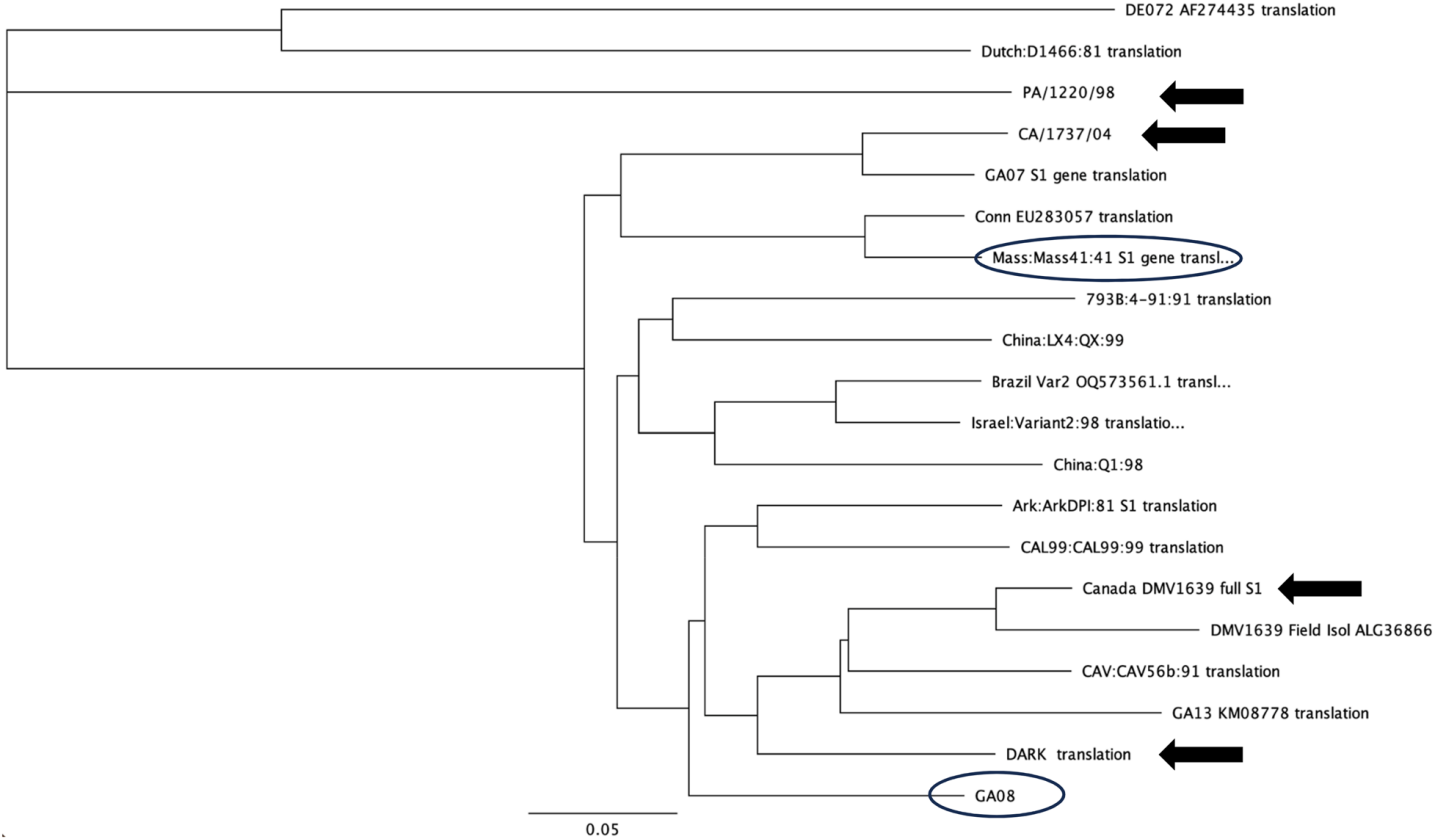
**Phylogenic tree (Neighbor-Joining tree building method, Geneious Prime) showing the relationship among the S1 glycoprotein amino acid sequences for the challenge viruses (arrows), the vaccines (circles) used in this study as well as reference strains (NCBI).**

Figure 1 shows the phylogenetic relationship among reference strains, and the viruses used in this study. Table 1 contains the percent similarities between the S1 glycoprotein amino acid sequences for the vaccine and challenge viruses.

**Table 1:** **S1 glycoprotein amino acid percent similarity of challenge viruses compared to the vaccines used in this study**

IBV strain	Vaccines	
	vGA08	Mass
NC/DARK/23	80.3%	76.0%
PA/1220/98	50.0%	49.4%
CA/1737/04	74.9%	78.5%
Canada/DMV1639/23	79.6%	73.8%

### Vaccine takes

At 5 dpv, the vGA08 vaccine virus was detected by RT-qPCR in 100% of the vaccinated birds and the Mass vaccine was detected in 92.6% of the vaccinated birds. All non-vaccinated birds were negative for vaccine virus at that time. At 26 dpv, birds were tested for vaccine virus utilizing the vGA08 and Mass type specific RT-qPCR assays and almost all of the vaccinated birds had essentially cleared the vaccine viruses with average CT values in the mid to upper 30’s. All non-vaccinated birds were negative. In addition, all vaccinated birds seroconverted at 26 dpv with a geometric mean titer of 2463 and a 47.2% coefficient of variation (IDEXX).

### Clinical signs

Table 2 shows the number of birds with moderate (score 3) to severe (score 4) respiratory signs in each group. Only one vaccinated bird challenged with NC/DARK/23 showed moderate clinical signs. All other vaccinated birds had no or mild respiratory signs. In addition, there was a statistical difference in clinical signs scores (Kruskal–Wallis test, *p* < 0.05) between vaccinated and non-vaccinated birds receiving the same challenge virus in the four tested strains.

**Table 2:** **Number of birds with moderate to severe clinical signs/total observed at 5 days post-challenge**

Treatment Groups	Challenge viruses			
	NC/DARK/23	PA/1220/98	CA/1737/04	Canada/DMV1639/23
Vaccinated^A^	1/10^B^	0/10	0/10	0/10
Non-vaccinated	5/10	5/9	4/10	10/10

^A^A full dose of vGA08 and Mass vaccine was given by eyedrop at one day of age

^B^The number of birds with moderate to severe respiratory signs (score of 3 or 4) per total in each group. Clinical signs were scored (1 = no outward signs; 2 = watery eyes or mucus in the nares; 3 = watery eyes and mucus in the nares and 4 = watery eyes and mucus in the nares and tracheal rales) and there was a statistical difference (*p* < 0.05) between vaccinated and non-vaccinated birds receiving the same challenge virus (Kruskal–Wallis test followed by Tukey multiple comparison test)

### Challenge virus detection

For the birds challenged with NC/DARK/23 and Canada/DMV1639/23, we used the DMV/1639 type specific RT-qPCR test, which allowed us to exclusively detect those challenge viruses. And, although the PA/1220/89 and CA/1737/04 challenge viruses were tested with the universal 5UTR RT-qPCR assay because type specific tests were not available, we predominately detected challenge virus in those groups because the presence of the vaccine viruses at that time (as indicated above) was negligible.

**Figure 2:**
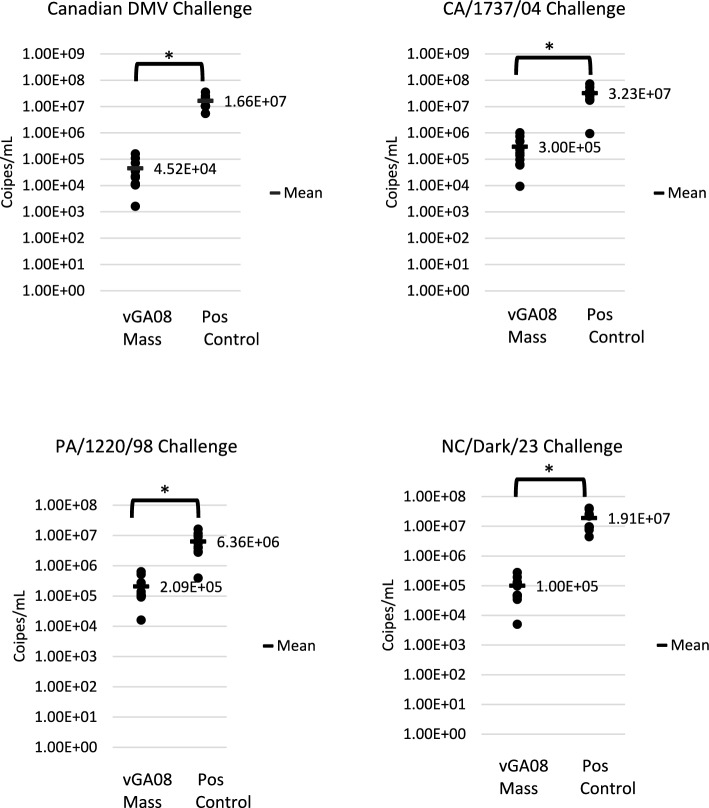
**Challenge virus detection (viral genome copies/mL) in choanal swabs at 5 days post-challenge**. Birds challenged with NC/DARK/23 and Canada/DMV1639/23, the DMV/1639 type specific RT-qPCR test was used. Birds challenged with PA/1220/89 and CA/1737/04 were tested using the universal 5UTR RT-qPCR test. * = statistically significant difference at *p* < 0.05 (Student’s *t*-test).

At 5 dpc, there was a statistically significant, (Student’s *t*-test, *p* < 0.05), reduction in the amount of challenge virus detected between vaccinated and non-vaccinated birds challenged with the same virus. A 2-log (100-fold) reduction was detected in the groups challenged with NC/DARK/23 and CA/1737/04. There was a 1-log (tenfold) reduction in the amount of challenge virus detected in the vaccinated group challenged with PA/1220/98 and there was a 3-log (1000-fold) reduction in the amount of challenge virus detected in the vaccinated group challenged with Canada/DMV1639/23 compared to non-vaccinated birds challenged with the same virus (Figure 2).

## Discussion

In this study, we found that simultaneous vaccination of SPF chicks at 1 DOA with vGA08 and Mass protected against challenge with either NC/DARK/23, PA/1220/98, CA/1737/04 or Canada/DMV1639/23 at 28 dpv. This protection was evident through mild to no clinical signs, and a 1–3 log reduction in the amount of challenge virus compared to non-vaccinated challenge controls.

The S1 glycoprotein amino acid sequence of the vGA08 vaccine used in this study was 50% to 80.3% similar to the challenge viruses, while the Mass vaccine was 49.4–78.5% similar to the challenge viruses. Conventional dogma suggests that a single IBV vaccine heterologous to the challenge virus would not protect. However, giving two or more different IBV vaccines simultaneously has been shown to provide cross-protection sufficient to protect against a heterologous challenge [13, 14]. Our results align with these reports.

Despite its benefits, heterologous cross-protection does not guarantee complete immunity. Birds can still become infected with the heterologous field virus and can exhibit mild clinical signs. Even homologous vaccination rarely induces sterilizing immunity, and high challenge loads in the field may still result is some mild clinical signs. Although not perfect, the heterologous cross-protection strategy can provide sufficient protection and safeguards against severe economic losses. It also provides a measure of safety against other IBV types that may unknowingly infect the birds.

Researchers have primarily demonstrated cross-protection by vaccination followed by heterologous challenge, with numerous reports in the literature [14–17]. Although the immune response to IBV has been studied, the exact mechanism underlying cross-protection remains unclear.

Several components of the immune response are thought to be involved in cross-protection. The innate immune response, including NK cells, toll-like receptors, interferon, complement and interleukin-1 beta play an important role in nonspecific immunity [18, 19]. In addition, both humoral and cellular adaptive immune responses, to B and T-cell epitopes on conserved viral proteins [20–22] and conserved regions on spike [23, 24] are believed to confer cross-protection [20, 25]. Furthermore, the cell mediated immune response, which is critical for controlling IBV infections in poultry, is inherently cross-reactive [26]. The cross-reactivity of cell mediated immunity stems from how cells process the viral proteins into small peptides and present them on the cell surface with the major histocompatibility complex (MHC) proteins. Indeed, cytotoxic lymphocytes recognizing epitope(s) on the nucleocapsid protein were cross-reactive with two different IBV strains [26].

Our study clearly shows cross-protection against IBV variant viruses currently circulating in North America. It is important to conduct these studies because cross-protection cannot be predicted from sequence data, nor does serology always correlate with protection [25]. Until the exact nature of a cross-protective immune response is elucidated, vaccine/challenge experiments similar to the one conducted herein are the only reliable method for assessing protection.

## Data Availability

All data generated and analyzed for this study are included in this published article.

## References

[1] Valastro V, Holmes EC, Britton P, Fusaro A, Jackwood MW, Cattoli G, Monne I (2016) S1 gene-based phylogeny of infectious bronchitis virus: an attempt to harmonize virus classification. Infect Genet Evol 39:349–364 26883378 10.1016/j.meegid.2016.02.015 PMC7172980

[2] Beckstead R, Brimer S, Callison S, Jackwood M. 2024. Identification of infectious bronchitis virus recombinants using next generation sequencing. International Avian Resp Dis Conf. Athens GA

[3] Kingham BF, Keeler CL, Nix WA, Ladman BS, Gelb J Jr (2000) Identification of avian infectious bronchitis virus by direct automated cycle sequencing of the S-1 gene. Avian Dis 44:325–335 10879913

[4] Jackwood MW, Hilt DA, Williams SM, Woolcock P, Cardona C, O’Connor R (2007) Molecular and serologic characterization, pathogenicity, and protection studies with infectious bronchitis virus field isolates from California. Avian Dis 51:527–533 17626478 10.1637/0005-2086(2007)51[527:MASCPA]2.0.CO;2

[5] Hassan MSH, Buharideem SM, Ali A, Najmudeen SM, Goldsmith D, Coffin CS, Cork SC, van der Meer F, Abdul-Careem MF (2022) Efficacy of commercial infectious bronchitis vaccines against Canadian Delmarva (DMV/1639) infectious bronchitis virus infection in layers. Vaccines 10:1194–1207 36016082 10.3390/vaccines10081194 PMC9416550

[6] Ali A, Ojkic D, Eishafiee EA, Shany S, El-Safty MM, Shalaby AA, Abdul-Careem MF (2022) Genotyping and in silico analysis of Delmarva (MDV/1639) infectious bronchitis virus (IBV) spike 1 (S1) glycoprotein. Genes 13:1617–1635 36140785 10.3390/genes13091617 PMC9498812

[7] National Library of Medicine. National Center for Biotechnology Information. https://www.ncbi.nlm.nih.gov/

[8] Geneious. https://www.geneious.com/

[9] Callison SA, Hilt DA, Boynton TO, Sample BF, Robison R, Swayne DE, Jackwood MW (2006) Development and evaluation of a real-time Taqman RT-PCR assay for the detection of infectious bronchitis virus from infected chickens. J Virol Methods 138:60–65 16934878 10.1016/j.jviromet.2006.07.018 PMC7112890

[10] Mo J, Angelichio M, Gow L, Leathers V, Jackwood MW (2020) Validation of specific quantitative real-time RT-PCR assay panel for infectious bronchitis using synthetic DNA standards and clinical specimens. J Virol Methods 276:113773 31712094 10.1016/j.jviromet.2019.113773 PMC7113781

[11] Jackwood MW, Rosenbloom R, Petteruti M, Hilt DA, McCall AW, Williams SM (2010) Avian coronavirus infectious bronchitis virus susceptibility to botanical oleoresins and essential oils in vitro and in vivo. Virus Res 149:86–94 20096315 10.1016/j.virusres.2010.01.006 PMC7114412

[12] Prism v6.0 software. Graphpad. https://www.graphpad.com/.

[13] Brimer SK, Fischer EAJ, Beckstead R, White J, Cazaban C, Tatar-Kis T, Velkers FC, El Attrache J, Stegeman A (2024) A vaccine program comprising GA08 (GI-27) and Mass (GI-1) strains prevents DMV1639 (GI-17) infectious bronchitis virus transmission among broiler chickens. Avian Pathol 54:83–95 39045705 10.1080/03079457.2024.2383765

[14] Cook JK, Orbell SJ, Woods MA, Huggins MB (1999) Breadth of protection of the respiratory tract provided by different live-attenuated infectioius bronchitis vaccines against challenge with infectious bronchitis viruses of heterologous serotypes. Avian Pathol 28:477–485 26911602 10.1080/03079459994506

[15] Bru T, Vila R, Cabana M, Geerligs HJ (2017) Protection of chickens vaccinated with combinations of commercial live infectious bronchitis vaccines containing Massachusetts, Dutch and QX-like serotypes against challenge with virulent infectious bronchitis viruses 793B and IS/1494/06 Israel variant 2. Avian Pathol 46:52–58 27400065 10.1080/03079457.2016.1203393

[16] Smialek M, Tykalowski B, Dziewulska D, Stenzel T, Koncicki A (2017) Immunological aspects of the efficiency of protectotype vaccination strategy against chicken infectious bronchitis. BMC Vet Res 13:44–50 28178957 10.1186/s12917-017-0963-1 PMC5299672

[17] Toro H, van Santen L, Ghetas AM, Joiner KS (2015) Cross-protection by infectious bronchitis viruses under controlled experimental conditions. Avian Dis 59:532–536 26629628 10.1637/11231-070615-Reg.1

[18] Gao Z, Hu J, Cai Y, Liu Y, Yin G, Guo X, Wang R, Zhong M, Liu Q, Feng X (2025) Identification of B-cell epitopes located on the surface of the S1 protein of infectious bronchitis virus M41 strains. Viruses 17:464–480 40284907 10.3390/v17040464 PMC12031124

[19] Yang X, Li J, Liu H, Zhang P, Chen D, Men S, Li X, Wang H (2018) Induction of innate immune response following introduction of infectious bronchitis virus (IBV) in the trachea and renal tissues of chickens. Microb Pathog 116:54–61 29330060 10.1016/j.micpath.2018.01.008

[20] Qin Y, Tu K, Teng Q, Feng D, Zhao Y, Zhang G (2021) Identification of novel T-cell epitopes on infectious bronchitis virus N protein and development of a multi-epitope vaccine. J Virol 95:e00667-21 34105997 10.1128/JVI.00667-21 PMC8354238

[21] Seah NJ, Yu L, Kwang J (2000) Localization of linear B-cell epitopes on infectious bronchitis virus nucleocapsid protein. Vet Microbiol 75:11–16 10865148 10.1016/s0378-1135(00)00202-9

[22] Yu D, Han Z, Xu J, Shao Y, Li H, Kong X, Liu S (2017) A novel B-cell epitope of avian infectious bronchitis virus N protein. Viral Immunol 23:189–199 10.1089/vim.2009.0094 20373999

[23] Eldemery F, Joiner KS, Toro H, van Santen VL (2017) Protection against infectious bronchitis virus by spike ectodomain subunit vaccine. Vaccine 35:5864–5871 28899630 10.1016/j.vaccine.2017.09.013 PMC7111290

[24] Zou N, Xia J, Wang F, Duan Z, Miao D, Yan Q, Cao S, Wen X, Liu P, Huang Y (2015) Two novel neutralizing antigenic epitopes of the s1 subunit protein of a QX-like avian infectious bronchitis virus strain Sczy3 as revealed using a phage display peptide library. Vet Immunol Immunopathol 168:49–55 26315775 10.1016/j.vetimm.2015.08.008 PMC7127571

[25] Cook JK, Jackwood M, Jones RC (2012) The long view: 40 years of infectious bronchitis research. Avian Pathol 41:239–250 22702451 10.1080/03079457.2012.680432

[26] Collisson EW, Pei J, Dzielawa J, Seo SH (2000) Cytotoxic T lymphocytes are critical in the control of infectious bronchitis virus in poultry. Dev Comp Immunol 24:187–200 10717287 10.1016/s0145-305x(99)00072-5

